# INTERVENTIONS FOR PERSONS WITH YOUNG-ONSET DEMENTIA AND THEIR FAMILIES: A SCOPING REVIEW

**DOI:** 10.1093/geroni/igad104.3440

**Published:** 2023-12-21

**Authors:** Xiaoyan Cui, Junqiao Wang, Bei Wu, Qianhua Zhao, Xueting Tang, Roy Thompson, Jing Wang

**Affiliations:** Fudan University, Shanghai, Shanghai, China (People’s Republic); Fudan University, Shanghai, Shanghai, China (People’s Republic); New York University, New York City, New York, United States; Fudan University Affiliated Huashan Hospital, Shanghai, Shanghai, China (People’s Republic); Fudan University, Shanghai, Shanghai, China (People’s Republic); University of Missouri, Columbia, Missouri, United States; University of New Hampshire, Durham, New Hampshire, United States

## Abstract

Young Onset Dementia (YOD) poses unique challenges for individuals and their families globally by impacting their working-age years and family responsibilities. The paucity of effective treatments emphasizes the need for tailored interventions and support services to enhance quality of life and social engagement. Dementia care services oftentimes fail to meet YOD-specific needs, necessitating customized approaches. This review aims to synthesize non-pharmacological intervention studies for YOD individuals and families to inform future targeted interventions. We conducted a systematic literature search across four databases. Included articles were carefully screened, categorized, and synthesized. This review examined 12 studies focused on persons with YOD. Interventions targeted daily life support, cognitive decline, and social engagement, using quantitative, qualitative, or mixed methods designs. Nine studies addressed interventions for families focused on self-efficacy, burden, anxiety, depression, quality of life, post-traumatic stress, and well-being. This review revealed a dearth of family-centered intervention studies, emphasizing the need for more comprehensive and tailored interventions for families of individuals with YOD. There is urgent need for more large-scale intervention studies, especially from Low and Middle income countries, which should adopt family-centered and life course perspectives. For future interventions , more extensive incorporation of stakeholder involvement is essential for successful implementation. Moreover, the integration of new technologies shows promise as a potential avenue for intervention advancement.

